# Mothers' AdvocateS In the Community (MOSAIC)- non-professional mentor support to reduce intimate partner violence and depression in mothers: a cluster randomised trial in primary care

**DOI:** 10.1186/1471-2458-11-178

**Published:** 2011-03-23

**Authors:** Angela J Taft, Rhonda Small, Kelsey L Hegarty, Lyndsey F Watson, Lisa Gold, Judith A Lumley

**Affiliations:** 1 Associate Professor, Mother and Child Health Research, La Trobe University, Melbourne, Vic 3000, Australia; 2 Professor, Mother and Child Health Research, La Trobe University, Melbourne, Vic 3000, Australia; 3 Associate Professor, Primary Care Research Unit, Department of General Practice, University of Melbourne, Parkville, Vic 3053, Australia; 4 Senior Research Fellow, Mother and Child Health Research, La Trobe University, Melbourne, Vic 3000; 5 Senior Research Fellow, Deakin Health Economics, Deakin University, Burwood, Vic 3125, Australia; 6 Emeritus Professor (Retired), Mother and Child Health Research, La Trobe University, Melbourne, Vic 3000, Australia

## Abstract

**Background:**

Effective interventions to increase safety and wellbeing of mothers experiencing intimate partner violence (IPV) are scarce. As much attention is focussed on professional intervention, this study aimed to determine the effectiveness of non-professional mentor support in reducing IPV and depression among pregnant and recent mothers experiencing, or at risk of IPV.

**Methods:**

MOSAIC was a cluster randomised trial in 106 primary care (maternal and child health nurse and general practitioner) clinics in Melbourne, Australia. 63/106 clinics referred 215 eligible culturally and linguistically diverse women between January 2006 and December 2007. 167 in the intervention (I) arm, and 91 in the comparison (C) arm. 174 (80.9%) were recruited. 133 (76.4%) women (90 I and 43 C) completed follow-up at 12 months.

*Intervention*: 12 months of weekly home visiting from trained and supervised local mothers, (English & Vietnamese speaking) offering non-professional befriending, advocacy, parenting support and referrals.

*Main outcome measures*: Primary outcomes; IPV (Composite Abuse Scale CAS) and depression (Edinburgh Postnatal Depression Scale EPDS); secondary measures included wellbeing (SF-36), parenting stress (PSI-SF) and social support (MOS-SF) at baseline and follow-up.

*Analysis*: Intention-to-treat using multivariable logistic regression and propensity scoring.

**Results:**

There was evidence of a true difference in mean abuse scores at follow-up in the intervention compared with the comparison arm (15.9 vs 21.8, AdjDiff -8.67, CI -16.2 to -1.15). There was weak evidence for other outcomes, but a trend was evident favouring the intervention: proportions of women with CAS scores ≥7, 51/88 (58.4%) vs 27/42 (64.3%) AdjOR 0.47, CI 0.21 to 1.05); depression (EPDS score ≥13) (19/85, 22% (I) vs 14/43, 33% (C); AdjOR 0.42, CI 0.17 to 1.06); physical wellbeing mean scores (PCS-SF36: AdjDiff 2.79; CI -0.40 to 5.99); mental wellbeing mean scores (MCS-SF36: AdjDiff 2.26; CI -1.48 to 6.00). There was no observed effect on parenting stress. 82% of women mentored would recommend mentors to friends in similar situations.

**Conclusion:**

Non-professional mentor mother support appears promising for improving safety and enhancing physical and mental wellbeing among mothers experiencing intimate partner violence referred from primary care.

**Trial registration:**

ACTRN12607000010493http://www.anzctr.org.au

## Background

Intimate partner violence (IPV) is a prevalent and pressing public health issue, especially among women with young children in socio-economically deprived communities[1]. IPV can be present prior to pregnancy, continue or commence in pregnancy or start in the postnatal period[2]. It is clearly associated with detrimental health effects on women, their children and families[1]. Isolation, maternal depression and parenting stress are common consequences for abused mothers[3,4].

Early government policy responses to IPV in Australia, as elsewhere in Western countries, prioritised crisis intervention from police, legal and domestic violence services. More recently, a fervent debate about health care professional IPV screening and early intervention has included a focus on the lack of evidence for effective interventions[5]. While professional intervention has been recently examined,[6] less research attention has been paid to the potential contribution of non-professional support for primary care patients.

Pregnant women and those with young children frequently attend primary care [7] General practice (GP) and maternal and child health (MCH) clinics (free community-based centres for women with children from birth to six years of age) are universal, accessible and affordable primary care services in Melbourne, Australia. Thus, there are opportunities for prevention and early intervention strategies offered by the less stigmatising, confidential environment available in primary care. However, clinicians are often hampered by a range of individual and contextual barriers[8,9]. Inadequate training and support for identifying and managing IPV is complicated by limited evidence for what subsequent interventions to which they could refer, are beneficial for women from primary care settings[10,11].

Peer support and home visiting strategies have been shown to reduce maternal depression in the postnatal period,[12-14] but the picture is less clear when IPV is present[15]. In the most recent Cochrane review of IPV advocacy interventions, Ramsay *et al *(2009) argued for an increased range of intervention studies overall, but in particular for women remaining at home with perpetrators[6].

The MOSAIC (MOtherS' Advocates In the Community) model trialled in the current study combined evidence for the benefits of social support,[16] advocacy,[17] and antenatal mentoring [18] to reduce partner violence and improve women's mental and physical health. We located the study in primary care to contribute to the limited evidence about effective referral and intervention strategies in this setting and because mothers experiencing IPV are more prevalent in these populations. The development of the study is outlined in more detail in the published protocol[19].

In a pragmatic cluster randomised trial, we tested the effectiveness of the model to reduce partner abuse and depression and improve women's health and attachment to their children among pregnant or recent mothers identified in primary care.

This paper reports on the primary outcomes of the study.

## Methods

**Figure 1:**
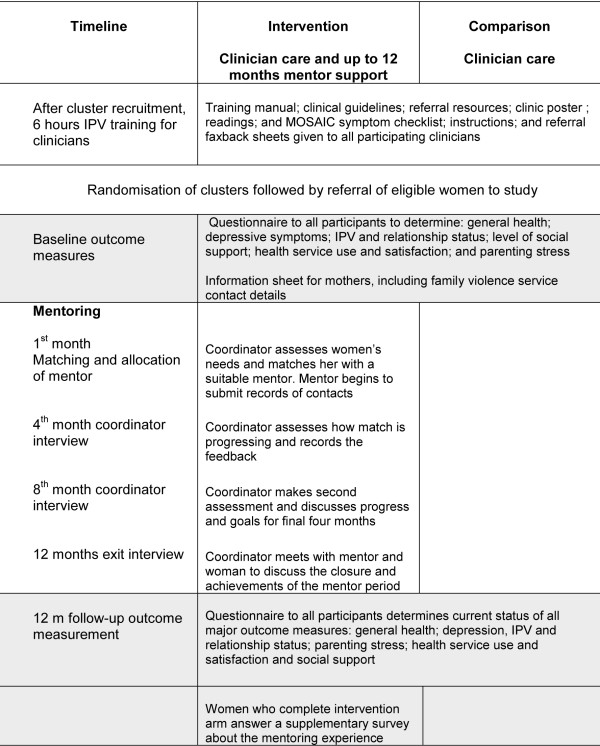
**MOSAIC implementation and timelines**.

Figure 1 graphically summarises the trial and intervention processes. More detail of clinician and participant recruitment, clinician training and ethics is provided in the published protocol[19].

### Cluster and clinician recruitment and randomisation

Seven hundred and ninety general practice clinics (one or two GPs) and 12 MCH teams (involving ~ 180 MCH clinics with one or two nurses) in the NW Melbourne region were eligible for recruitment. We targeted those GP clinics which offered shared care (care for pregnant patients shared between local GP and maternity hospital). Twenty-four practices (27 GPs) and eight MCH teams (82 clinics) were recruited and signed a memorandum of understanding to participate in IPV training and subsequent randomisation. A bilingual Vietnamese coordinator recruited Vietnamese practices (n = 4) for a Vietnamese sub-study. All participating clinicians undertook six hours of professional development to enhance their capacity to identify, respond to and refer women psychosocially distressed (at-risk) or experiencing IPV to community based services. Training for both MCH nurses and GPs was based on adult learning principles and contained both interactive and didactic elements. Clinicians were also provided with: referral booklets for IPV services; posters for waiting rooms; pocket-sized cards for women with local referral options and opportunities for further training.

After training, clinic randomisation occurred at two public meetings to which clinicians were invited. Sealed opaque envelopes contained the randomisation possibilities and a guest external to the project made the selection. GP clinics were randomised by number of GPs participating (one or two). MCH clinics were randomised by team to avoid contamination within teams and teams were stratified by numbers of births per local government area.

Given the cluster randomisation and the integration of the mentor model with enhanced primary care, clinicians were not blinded, nor were research staff, who fully briefed participants in the intervention arm about the mentoring program, negotiated informed consent and collected follow-up data about mentoring from participants in the intervention arm.

### Mentor recruitment and training

Following the appointment of two mentor coordinators, we advertised widely in local newspapers, schools and radio (Vietnamese) for women from the NW Melbourne suburbs to become mentors. The major criterion was that mentors be mothers with good listening skills and an open, compassionate and non-judgmental approach. Potential mentors phoned and applied in writing, were interviewed twice by coordinators, provided three references and a police check. MOSAIC provided initial five-day funded training that included befriending, domestic violence advocacy, working with depression, parenting support, safety and self care and then mentors met at regular intervals for further training and support. The MOSAIC training manual provides details of the mentor recruitment, training and support processes and is available online[20].

### Participant referral and recruitment

Women aged 16 and over attending GPs or MCH nurses were eligible to be referred to the study if they were pregnant or had at least one child five years or younger, and disclosed IPV or were psychosocially distressed. Psychosocial distress included women who had not disclosed but whose symptoms (depression, anxiety, frequent attendance without obvious cause etc) were indicative of abuse. Women were ineligible if they had a serious mental illness and were not taking medication, or their English was inadequate for informed consent, except for Vietnamese women, as Vietnamese bilingual staff and mentors participated in a sub-study.

From Jan 2006 to Dec 2007, clinicians were asked to identify consecutive eligible women and invite their participation in a study of enhanced primary care for mothers' emotional health. They requested and then faxed safe contact details of women willing to consider participation. Research staff then contacted women and visited at a safe time and place, provided more information, negotiated consent and gave all women a resource card for new mothers, which included contacts for family violence services[19]. Mentoring then commenced in the intervention arm (see Figure 1). Ethics approval was received from the Human Ethics Committees at both La Trobe University (03-76) and the University of Melbourne (030441).

### Objectives

The primary aims of the trial were:

• to reduce IPV and/or depression among women pregnant and/or with children under five whom GPs or MCH nurses identify as abused or at risk (psychosocially distressed); and

• to strengthen the general health and wellbeing and mother-child bonding of abused or at-risk women.

### Main outcome measures

We measured IPV using the Composite Abuse Scale (CAS), a well-validated and comprehensive measure[21]. An accepted cut-off score of ≥7[5] was used to indicate IPV. Maternal depression was assessed as a score of ≥13 on the EPDS, now validated for use outside the immediate postnatal period[22,23]. General health and wellbeing were assessed with the SF-36[24], and mother-child bonding expressed as parenting stress and attachment (using the attachment sub-scale) with the Parenting Stress Index Short form (PSI-SF)[25]. Social support (a potential effect modifier) was assessed with the Medical Outcomes Scale Short Form (MOS-SF)[26]. These measures were all included in the baseline and 12 month follow-up questionnaires, combined with questions about women's use of and satisfaction with their primary care services.

Resources used for training and support of clinicians and mentors and in mentor support of women were measured from study records and time use logs kept by research staff and mentors. Costs from a health sector perspective of women's use of health care services (including the intervention) over the 12 month period of the intervention were estimated using standard unit costs and presented in A$ 2009.

### Sample size

MOSAIC aimed to detect a difference of 16% (an improvement for one in six women was considered clinically important) in IPV or depression a year after recruitment with traditional levels of 80% power and 95% confidence. This was estimated to require 165 women in each trial arm with individual randomisation, from a level of any prevalence value for either IPV or depression between 30% and 70% in the control group. With cluster randomisation, assuming an intra-class correlation of 0.02 (previously found in a GP partner violence study [27], the sample size required increased to 190 in each arm. Given the mobility of this vulnerable, sometimes fearful population we conservatively estimated an attrition and loss rate of as much as 45%, requiring at most 350 to be recruited in each arm.

Due to lower than expected numbers of women referred and recruited by the end of the trial, we estimated prior to data analysis that the achieved sample size would have 80% power and 95% confidence to detect a reduction of 22% for IPV, 18% for depression and a difference of two units in the mental component score (MCS) of the SF36.

### Documenting the intervention for process and impact evaluation

Comprehensive process evaluation is detailed in the protocol[19]. This included: interim and impact surveys of participating GPs and nurses; fortnightly mentor contact sheets; four, eight and twelve month (exit) coordinator interviews with participants; and a supplementary impact questionnaire for intervention participants about the experience of being mentored (undertaken at the same time as the follow-up survey was completed). Qualitative semi-structured interviews with a diverse sample of mentored women (n = 35) about their experiences and also of their mentors' experiences (n = 15) were undertaken and will be published separately.

An economic cost-consequences analysis was also conducted.

### Analysis

Descriptive statistics of the characteristics of the intervention vs. comparison groups at recruitment were calculated to check that cluster randomisation resulted in similar groups. The main analysis was conducted as intention-to-treat and included a comparison of the primary outcomes pre- and post intervention. These pre-specified measures are the proportion and means of those experiencing abuse (CAS≥7), depression (the proportion with a score ≥13 and mean EPDS score), mean scores on the Mental and Physical Health Component Scores of the SF36, the mean score on the Parenting Stress Index and the mean subscale on parent-child interaction.

Due to an imbalance in the numbers of women recruited in the two arms of the trial, a propensity score (PS) analysis was also undertaken to balance the arms for potential confounding from possible selection bias,[28,29] after multiple imputation[30] for missing data using the ICE and MIM routines in Stata10[31].

In the outcome models, the intervention effect was estimated adjusting for the baseline measure of each outcome variable as possible effect modifiers. Results are presented before and after inclusion of the propensity score that adjusts for possible selection bias. Multivariate logistic regression analyses were carried out using STATA 10,[31] and all were adjusted for the cluster design.

## Results

**Figure 2:**
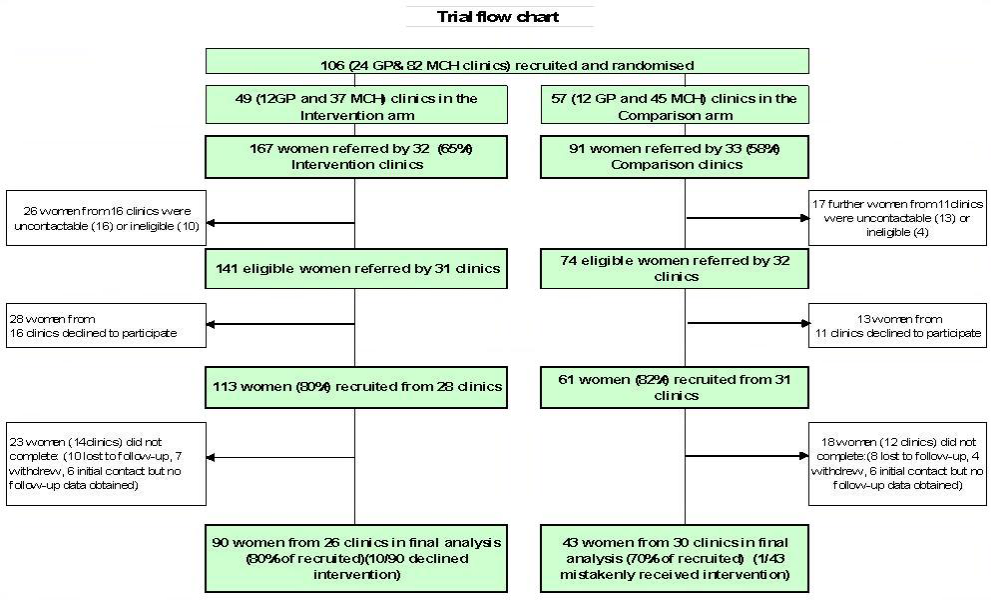
**Trial flow chart**.

Figure 2 describes sample recruitment and retention in the trial. Over the two years of recruitment, clinicians from 65/106 (59.4%) centres identified and referred 258 women, of whom 215 (167 women in the intervention and 91 in the comparison arm) were eligible. Similar proportions of GPs (46%) and nurses (51%) referred no women at all, and only 7% of nurses and 11% of GPs referred 6 or more women (not shown). MOSAIC recruited 174/215 (82%) of the eligible women referred to the study (113 intervention and 61 comparison) and retained 76% (of those recruited) at twelve months (133/174). Thus, 62% of referred and eligible women were both recruited to, and completed the study (133/215). This consisted of 90 women in the intervention arm and 43 in the comparison arm. All women were included in the intention to treat analysis, even though, as often in pragmatic trials, 10 women refused the intervention and one woman in the comparison arm mistakenly received it.

### Baseline characteristics

Table 1 compares the characteristics of women recruited and retained in the trial with those subsequently lost to follow-up. At baseline, there were few differences in the socio-demographic profiles of participants retained in the study. Both groups included a high proportion of disadvantaged women, compared with non-Aboriginal Australian women giving birth in Victoria [32]. Around 70% in both arms had a health care card (i.e. a subsidy for those with inadequate income) compared with 22% of Victorian women aged 15-44 [33], over half were dependent on a pension or benefit and over a third were born overseas, compared with 24% in Victoria.

**Table 1:** Characteristics of women retained and those lost to follow up at baseline by allocation status

	Women retained				Women lost to follow-up	
	Intervention n = 90 (%)	Comparison n = 43 (%)			Intervention n = 23 (%)	Comparison n = 18 (%)
Age						
Mean (SD)	32.0 (6.7)	32.4 (5.8)			32.6 (6.1)	29.6 (6.1)
Marital status						
Married	29 (32)	11 (26)			6 (26)	4 (22)
De Facto	18 (20)	10 (23)			3 (13)	5 (28)
Divorced/separated	22 (24)	10 (23)			6 (26)	4 (22)
Single/not living with partner	20 (22)	11 (25)			8 (35)	5 (28)
Number of children						
One	41 (46)	23 (53)			6 (26)	7 (39)
Two or more	48 (54)	20 (46)			17 (74)	11 (61)
Education level attained						
Year 12 or less	43 (47)	23 (51)			13 (56)	10 (56)
Healthcare card						
Yes	67 (74)	30 (70)			18 (78)	13 (72)
Income						
Pension/welfare	56 (62)	23 (53)			14 (61)	11 (61)
Country of birth						
Overseas born	32 (36)	14 (32)			5 (22)	10 (56)

			P-value			
Partner Abuse (CAS)				(+PS adj)		
Score 7+	71 (79)	32 (74)	0.8	1.0	22 (96)	16 (89)
Mean (SD)	22.5 (23.0)	23.6 (19.0)			41.7 (30.2)	17.1 (11.3)
Median (Interquartile range)	19 (7-31)	18 (10-37)			32 (18-56)	13 (10-23)
						
Depression (EPDS Score 13+)						
Yes	63 (70)	25 (58)	0.2	0.7	18 (78)	8 (44)
						
SF36						
MCS Mean (SD)	28.0 (9.9)	31.1 (8.1)	0.06	0.6	23.1 (10.6)	31.3 (12.6)
PCS Mean (SD)	49.0 (10.6)	48.1 (8.6)	0.6	0.9	53.4 (9.5)	48.3 (9.9)
Social Support						
Mean (SD)	2.84 (0.94)	3.28 (0.94)	0.01	0.4	2.58 (0.83)	3.06 (1.14)
Parenting Stress						
No. stressed (%)	40 (49)	13 (33)	0.08	0.7	18 (78)	12 (67)

Abbreviation: +PS adj = adjusted for Propensity Score

At baseline, measures of abuse were similar between groups[19], but the intervention group displayed higher levels of probable depression and parenting stress and significantly lower social support. Common to IPV studies[5], women lost to the study were more likely to be more severely abused.

### Intervention fidelity and women's feedback about the intervention

Of the 90 retained women randomised to the intervention, 86 completed supplementary questions about their mentoring experiences. Of these, 10 women declined mentoring due to lack of time or perceived need and 76 women received some mentoring support. 58/76 (76%) received 12 months mentoring and 10.5% (8/76) between three and nine months. The remainder were mentored for less than three months. A majority of women (58%) reported meeting weekly with their mentor, 18% fortnightly and 20% reported no regular pattern. Women met with their mentor in the woman's home (57%), or elsewhere (26%). Most women (87%) reported that the time they spent was just right, with 10.5% believing it was not enough and 2.5% that it was too much.

Women reported being offered information most often about legal, self-care and parenting services (Table 2) but self care, educational, parenting and legal services were those that women reported they had most often actually used. Twice the proportion of mentored women 29/90 (32%) had taken up new studies or training or returned to further training or education over the past 12 months compared with non-mentored women - 7/43 (16%) (OR = 2.4, CI 1.08 to 5.02).

**Table 2:** Services women report their mentors offered, and services women reported that they used in the MOSAIC study

Type of service	Offered (%)	Used (% of offered)
	*(n = 76)	
Parenting support	34 (45)	14 (41)
Self-care services (exercise, yoga etc)	35 (46)	15 (43)
Educational support	19 (25)	7 (37)
Legal support	37 (49)	13 (35)
Financial help	15 (20)	6 (40)
Services for men who abuse partners	6 (8)	1 (17)
Services for children experiencing difficulties	14 (18)	5 (36)
Housing support	11 (15)	7 (64)
DV or FV services	25 (33)	9 (36)
Therapy or mental health services	16 (21)	6 (38)
Immigration or refugee services	5 (7)	2 (40)
Language classes	7 (9)	2 (29)
Other	2 (3)	2 (100)
Did not need or want to use services	9 (12)	

* 86/90 women in the intervention arm completed the supplementary survey reporting their mentoring experiences, however 10 women did not want a mentor

Percentages do not add to 100 as women could nominate more than one service if they were offered or used more than one

### Changes in primary and secondary outcome measures

Table 3 outlines the analysis of observed differences in means and proportions between the intervention and comparison arms for all outcomes, with adjusted odds ratios with and without propensity scores (PS). Only one outcome (mean CAS score differences) provided evidence of a true difference in abuse values. All other outcomes, with or without propensity scores, offered weaker or no evidence of a true difference, while indicating a trend in the directions expected.

**Table 3:** Partner abuse, depression, wellbeing, parenting stress and social support at follow-up adjusted for baseline scores and propensity scores

Outcome	Baseline Means or percentages		Follow-up Means or percentages		AdjOR (95% CI)	AdjDiff (95% CI)	P value	ICC adj	AdjOR (95% CI)	AdjDiff (95% CI)	P value	ICC adj
									
	Intervention	Comparison	Intervention	Comparison					+ adj for propensity score			
	(n = 90)	(n = 43)	(n = 90)	(n = 43)								
**Partner abuse**.	90	40	88	42								
Women abused, No. (% CAS≥7)	71 (79)	32 (80)	51 (58)	27 (64)	0.47 (0.21,1.05)		0.06	0	0.58 (0.21,1.58)			
CAS total score (SD)	22.5 (23.0)	23.6 (19.4)	15.9 (16.7)	21.8 (21.2)		-8.67 (-16.2,-1.15)	0.03	0		-8.75 (-18.2,0.70)	0.07	0.07

**Depressive** **symptoms**	89	42	85	43								
EPDS, No. (% ≥ 13)	63 (72)	25 (60)	19 (22)	14 (33)	0.42 (0.17,1.06)		0.07	0.08	0.72 (0.24,2.13)		0.5	0
EPDS mean score (SD)	15 (5.7)	12.9 (6.0)	8.9 (5.0)	9.9 (6.3)		-1.9 (-4.12,0.32)	0.09	0.04		-1.92 (-4.25,0.41)	0.11	0.14

**Wellbeing - SF36**	87	43	85	43								
Mental health mean score (SD)	28.0 (9.9)	31.1 (8.1)	38.4(10.4)	37.6 (10.8)		2.26 (-1.48,6.00)	0.2	0		3.42 (-0.52,7.37)	0.09	0
Physical health mean score (SD)	49.0 (10.6)	48.1 (8.6)	51.9 (11.9)	47.9 (8.0)		2.79 (-0.40,5.99)	0.09	0		2.14 (-2.07,6.36)	0.3	0.10

**Parenting stress**	81	40	87	42								
Parental distress, No. (%)	68 (78)	28 (65)	43 (49)	21 (49)	0.6 (0.32,1.49)		0.3	0	0.82 (0.34,2.01)		0.7	0
Parent-child dysfunctional interaction, No.(%)	26 (30)	7 (16)	24 (27)	9 (21)	1.0 (0.44,2.71)		0.8	0	1.16 (0.88,3.49)		0.8	0
Total parenting stress, No. (%)	40 (49)	13 (33)	39 (45)	15 (36)	1.0 (0.45,1.49)		0.9	0	0.86 (0.32,2.33)		0.8	0

**Social support** **(MOS) scores** **(range 0-5)**	80	42	81	39								
Mean score (SD)	2.84 (0.94)	3.28 (0.94)	3.29 (1.06)	3.45 (0.95)		-0.21 (-0.82,0.40)	0.5	0		-0.29 (-0.91,0.34)	0.4	0

#### Partner violence (Composite Abuse Scale)

The adjusted difference in the total CAS score from baseline was greater in the mentored compared with the non-mentored arm (Adj Diff = -8.67, range -16.2 to -1.15), but the evidence for this difference was weaker after PS adjustment. The odds of experiencing violence at follow-up, adjusted for baseline abuse were 0.47 (95%CI 0.21-1.05).

#### Depression (Edinburgh Postnatal Depression Scale)

Observed reduction in depression mean scores from 15.0 to 8.9 in the intervention arm compared favourably with non-mentored women (12.9 to 9.9), but the adjusted difference (AdjDiff) of -1.90, 95% CI -4.12 to 0.32, did not reach conventional statistical significance. The reduction from 72% to 22% of mentored women scoring as depressed compared with 60% to 33% in non-mentored women AdjOR 0.42, 95%CI 0.17 to 1.06).

#### General health and wellbeing (SF-36)

There was weak evidence for a difference between the intervention and comparison arms in general wellbeing improvements at follow-up, while mean adjusted differences on both the Mental and Physical Components Scores (MCS and PCS) favoured the intervention arm.

#### Parenting stress and parent child dysfunction (Parenting Stress Index)

The proportion of women experiencing parenting stress did not appear to be affected by the intervention.

#### Costs

There were no significant differences in use of health care services between groups over the 12 month follow-up period. Health sector costs were A$ 5,738 (US$5,083) per woman higher in the intervention group. However, this estimate is arguably inflated by the artificial trial setting, as 60 mentors were trained but only 32 provided mentoring (≥1 women). At a predicted mentor capacity of 4 women per year with biannual training, predicted costs would be A$2,313 (US$2,049) per woman.

## Discussion and Conclusion

The evidence for effective interventions to reduce IPV and improve abused women's wellbeing is very limited, especially for women still living with partners, which includes half the participants in this study. In this first primary care randomised trial of non-professional mentor support for women abused by intimate partners, there was evidence of a true difference of reduced partner violence between mentored women referred from primary care populations compared with those not mentored (although PS analysis weakened the evidence). There was weak evidence for other findings suggestive of mentor benefit in reducing depression and improving physical and mental wellbeing.

All findings were consistently in favour of the intervention arm, and the lack of stronger evidence for the differences may be due in part to the smaller than anticipated numbers of participants, resulting in reduced power to detect small, but meaningful differences. Despite substantial efforts to enhance clinician IPV knowledge, skills and resources, and clinician agreement to the randomised trial design, MOSAIC suffered from low rates of identification and referral from both nurses and doctors, particularly in the trial's comparison arm. There is evidence from clinician interviews of two problems. The first is that despite considerable training, many clinicians continued to feel under-confident to ask about IPV. We conclude that major systemic challenges remain to be overcome before health care providers feel sufficiently supported and confident to identify and effectively care for the abused women in their respective populations. Second, there was clearly a reluctance to refer women in the comparison arm in comparison with the intervention arm, due to a perception of no benefit for this group of vulnerable women even after feedback of positive comments about participation from women recruited to the comparison arm. This may also contribute to bias.

Thus, the major threat to the validity of the study was clinician non-blinding[34]. This aspect of the design was thought to be unavoidable, as the mentor-mother program was designed to be integrated with primary care and women did talk about mentoring with their clinician. Low referrals overall and the 2:1 ratio of recruited women in the intervention versus comparison arms, introduced potential selection bias and had a substantial impact on our final sample size and our power to provide better evidence for intervention effects. Our approach to addressing selection bias involved adding PS to our analysis. While the PS analysis did result in altered effect sizes, it did not alter the direction of effects. The PS analysis did not alter the consistent trend towards more favorable outcomes in the intervention arm of the trial.

Could we have avoided the problem? One solution would have been to randomise individual women once they had been referred to the research team. With hindsight this has some merit, given the small number of women any individual clinician actually referred, which meant that contamination may have been unlikely. However, it is also unclear whether the trial would have experienced even lower numbers of referrals if clinicians had known that women referred would only have a 50:50 chance of receiving the offer of a mentor.

The self-report nature of the measures and the fact that we measured outcomes immediately after the intervention was completed, may be additional sources of bias. It remains unknown if any beneficial effect was sustained beyond 12 months.

In the only other mentoring study - *Madres a Madres*, a study among an abused pregnant population where the majority of mentoring was provided by telephone and only up until the time of birth-McFarlane *et al *found that the benefits of mentoring during pregnancy were not sustained to 18 months after birth[35,18]. Birth and infancy are periods of considerable strain among families where there is no IPV, so the additional stress for mothers experiencing IPV is self-evident, whenever the abuse commenced. The emotional and practical support from mentors during this infancy period, sustained for up to a year as in the current study, may explain why our findings suggest some benefit.

Our study findings are consistent with the growing number of studies providing evidence of the benefit of home-visiting for vulnerable mothers[36-38]. Yet, in the great majority of these, home visiting is provided by nurses and there is no evidence to date that nurse visits are effective when there is IPV present[15]. Nurses are often mandated to report child abuse and many women affected by IPV are fearful of losing their children or have many professionals already involved in their care. Given the findings of the current study we suggest there is a role for non-professional befriending models in the spectrum of professional and non-professional responses to IPV.

Further research is needed to confirm the findings of MOSAIC and to determine whether positive effects can be sustained over the longer term. MOSAIC will contribute to the small but increasing trial evidence for advocacy interventions for women in health care settings, demonstrated in the recent Cochrane systematic review[10]. Given the problems experienced in the current study with clinician identification and referral, despite significant investment in upskilling and support resources, the question of what more can be done to enhance clinician care when IPV is present - for women, their children and in management of abusing partners - requires further investigation.

## Competing interests

The authors declare that they have no competing interests.

## Authors' contributions

AT had full access to all of the data in the study and takes responsibility for the integrity of the data and the accuracy of the data analysis. AT was responsible for the study concept and study supervision. AT, RS, KH, LW, JL designed the overall study. LG designed the economic evaluation. LW, AT, RS, KH analysed the data and LG the economic evaluation. AT, LW, RS and LG drafted and were responsible for critical revision of the manuscript for important intellectual content. All currently employed authors (AT, RS, KH, LW, LG) reviewed and approved the final manuscript.

## Pre-publication history

The pre-publication history for this paper can be accessed here:

http://www.biomedcentral.com/1471-2458/11/178/prepub
